# The Silicea Headache

**Published:** 1882-11

**Authors:** Carroll Dunham


### SILICEA HEADACHE.

The headache produced by Silicea is so characteristic and well-defined, and, withal, corresponds to a form so frequently met with, that it is one of the remedies most frequently employed by me for headache. The pain involves the occiput, nape of the neck, vertex and the eyes, or generally the right eye.

It is a sticking or tearing, pressing pain, generally beginning in the neck and shoulders and going upward to the occiput and vertex, and extending through the head to the right eye.

Its conditions are characteristic, for it is much aggravated by motion, noise or light, the senses of sight and hearing being unnaturally acute. The patient prefers to lie down in a dark, quiet room. It is relieved by warm applications to the head. When most violent, it is accompanied by nausea and vomiting, and it passes away during sleep. The face is pale. In its conditions of aggravation this headache resembles that of Spigelia, but the latter affects the left eye and temple rather than the right, and is not relieved by warmth; but it is mitigated by pressure, and the pain does not come from the neck and shoulders.

The headache of Paris quad. (a valuable remedy in headache) has some resemblance to that of Silicea. The sensation is, however, a kind of tightness, as if the cerebral membranes were on the stretch, with pressure on the temples and very painful, as though a cord were stretched tightly from the back of the eyeballs to the centre of the brain. The headache is aggravated by thinking and relieved by pressure. The eyeballs feel too large for the orbits.

The headache of Menyanthes is a pressure from above downward; or, in the forehead, from within out; or, in the temples, a lateral inward pressure, with pressure in the eyeballs. It is relieved by compression of the head, but neither this nor the other remedies, except Silicea, has mitigation from warmth. I mention these remedies (Paris quad. and Menyanthes) because, like Silicea, I believe they are not so frequently used in treating headaches as they might be, with advantage. CARROLL DUNHAM.

